# Purtscher Retinopathy Resulting from a Car Crash Accident—Multimodal Imaging Value

**DOI:** 10.3390/diagnostics14141476

**Published:** 2024-07-10

**Authors:** Grzegorz Rotuski, Magdalena Bojarska, Michał Patyk, Radosław Różycki, Joanna Gołębiewska

**Affiliations:** Department of Ophthalmology, Military Institute of Aviation Medicine, 01-755 Warsaw, Poland; mbojarska@wiml.waw.pl (M.B.); mpatyk@wiml.waw.pl (M.P.); rrozycki@wiml.waw.pl (R.R.); jgolebiewska@wiml.waw.pl (J.G.)

**Keywords:** Purtscher retinopathy, Purtscher-like retinopathy, Purtscher flecken, traumatic retinopathy, multifocal electroretinography, visual evoked potentials, kinetic perimetry, OCT, fluorescein angiography

## Abstract

Purtscher retinopathy is a rare but severe sight-threatening eye condition that mostly occurs in middle-aged men after chest compression or head injury. In cases such as acute pancreatitis, connective tissue disorders, kidney failure or COVID-19 infection with similar ocular findings but no history of trauma, a diagnosis of Purtscher-like retinopathy is made. We present a case of a 72-year-old female with typical symptoms of Purtscher retinopathy in both eyes after a car crash accident. Although the pathophysiology of the disease is not fully understood, the main cause of Purtscher retinopathy seems to be an embolic occlusion of the precapillary arterioles which supply the superficial peripapillary capillaries. Activation of the C5a component of the complement predisposes the leukocytes to aggregation, which obstructs blood flow. The main symptom of Purtscher retinopathy is sudden, painless deterioration of vision which occurs up to 48 h after the injury. In most patients, the changes observed in the fundus of the eye resolve within several months, and visual acuity slowly improves, sometimes even returning to the state from before the injury. However, risk factors such as older age, high hyperopia, and late treatment implementation can make the prognosis less favorable.

The pathognomonic symptom of the Purtscher retinopathy is Purtscher flecken, described as multiple polygonal patches of intraretinal whitening with a clear zone of 50 microns on either side of the retinal vessels (arterioles or venules) [1,2,3]. The ischemic lesions are mainly restricted to the posterior pole. The annual incidence of symptomatic Purtscher retinopathy has been estimated to be 0.24 cases per million individuals in a population. The disease occurs bilaterally in 60% of cases [4]. Visual acuity initially progresses from counting fingers to 20/200, with potential improvement to 20/30 over a period of many months. Causes that need to be taken into consideration in the differential diagnosis of Purtscher retinopathy include the following: traumatic events (head contusion, chest compression, fracture of long bones, orthopedic surgery, extreme weightlifting, shaken baby syndrome, barotrauma, orbital injection of steroids, retrobulbar anesthesia, etc.), and non-traumatic causes (acute pancreatitis, chronic kidney disease, connective tissue diseases, blood clotting disorders, embolism, preeclampsia) [5]. 

A 72-year-old female presented to the Ophthalmology Department 7 days after a traffic accident. The ophthalmologic examination revealed the best corrected visual acuity (BCVA) of counting fingers (CF) in both eyes. Intraocular pressure in both eyes remained within normal range. The anterior segment evaluation did not reveal any abnormalities. The patient was admitted to the ward for prompt intravenous infusion of methylprednisolone, pentoxifylline, and fluid therapy. Additionally, she received dexamethasone and nepafenac eyedrops, antihypertensive drugs, and oral acetazolamide to improve the blood supply to the posterior pole of the eyeball. She was also administered with low-molecular-weight heparin and vitamins (B1, B6, B12) subcutaneously daily. Fluorescein angiography (Figure 1B) performed on the next day after the patient’s admission to the Ophthalmology Department demonstrated bilateral hypoperfusion of the posterior pole with subtle vessel leakage and hyperfluorescence of the optic disc margin in the temporal quadrant. On day 4 of hospitalization, a significant improvement in retinal morphology was observed, but visual acuity was still at the level of counting fingers at a slightly greater distance. After being discharged, she was switched to oral steroids, which decreased gradually in time, and then the supplementation of citicoline 500 mg daily was added. Follow-up at 1 month (Figure 1C) illustrated major resolution of pathological changes in time. Already after 2 months, the eye fundi looked almost normal (Figure 1D). At 6 months, the view was slightly opaque because of the accelerated development of cataract (Figure 1E). The final BCVA of the patient was 20/160 in the right eye and 20/63 in the left eye. The patient was also monitored using perimetry and optical coherence tomography (OCT) (Figure 2 and Figure 3). OCT-Angiography did not reveal any relevant abnormalities in the superficial and deep plexuses, nor in the choriocapillaris layer throughout the observation period. Multifocal electroretinography and visually evoked potentials were additionally performed (Figure 4).

According to evidence-based medicine, the mainstay of treatment is observation due to the self-limiting nature of Purtscher retinopathy. Some practitioners opt for rapid administration of intravenous steroid pulses, but researchers postulate that there is no significant difference in eyesight recovery regardless of their implementation [6,7,8]. Steroids aim to stabilize the membranes of neurons and microvascular channels, thereby limiting the release of pro-inflammatory factors and the accumulation of neutrophils, along with the activation of the complement system, resulting in apoptosis of retinal cells. However, there is a lack of reliable studies assessing their actual effectiveness compared to their omission. The age of patients may be important, as younger people have better regenerative potential, but they also tend to have a stronger inflammation response. Careful differentiation from Purtscher-like retinopathies is crucial, as the use of steroids alone when the underlying cause is acute pancreatitis or paraneoplastic syndrome may be associated with a worse prognosis than usual observation [9]. Additionally, the use of fluid therapy may be beneficial, especially in the case of post-traumatic hypovolemia, as well as to filter the embolic material and provide proper nutrition to the metabolically demanding retina. The use of agents that improve eye microcirculation, such as pentoxifylline or carbonic anhydrase inhibitors, may also be helpful. To reduce the risk of thrombosis, it is worth using anticoagulants and antiplatelet treatment. If macular edema or late retinal neovascularization occurs several months after the event due to chronic ischemia, intravitreal anti-VEGF injections are recommended [10,11]. Other potential therapies are based on isolated reports. A case of oral treatment with indomethacin 25 mg daily and papaverine for 6 weeks was described, with improvement in BCVA vision from counting fingers to 6/12 [12]. Another patient, after a chest compression injury, was subjected to hyperbaric oxygen therapy (39 sessions at 2 atm for 90 min twice a day), also with a final improvement in vision after 3 months from CF from 1 m to 8/10 and HM to CF from 50 cm [13]. In turn, following the theory of thrombotic microangiopathy, the use of bortezomib may be justified but needs further studies. It is a 26S proteasome inhibitor, used mainly in the treatment of hematological malignancies, but also works by inhibiting the activity of ADAMTS-13 metalloproteinase, also known as von Willebrand factor (vWF) cleavage protein, contributing to improve microcirculation [14]. Nevertheless, the outcome of the case reports with treatment is not superior to cases with sole observation, hence overtreatment should be avoided.

## Figures and Tables

**Figure 1:**
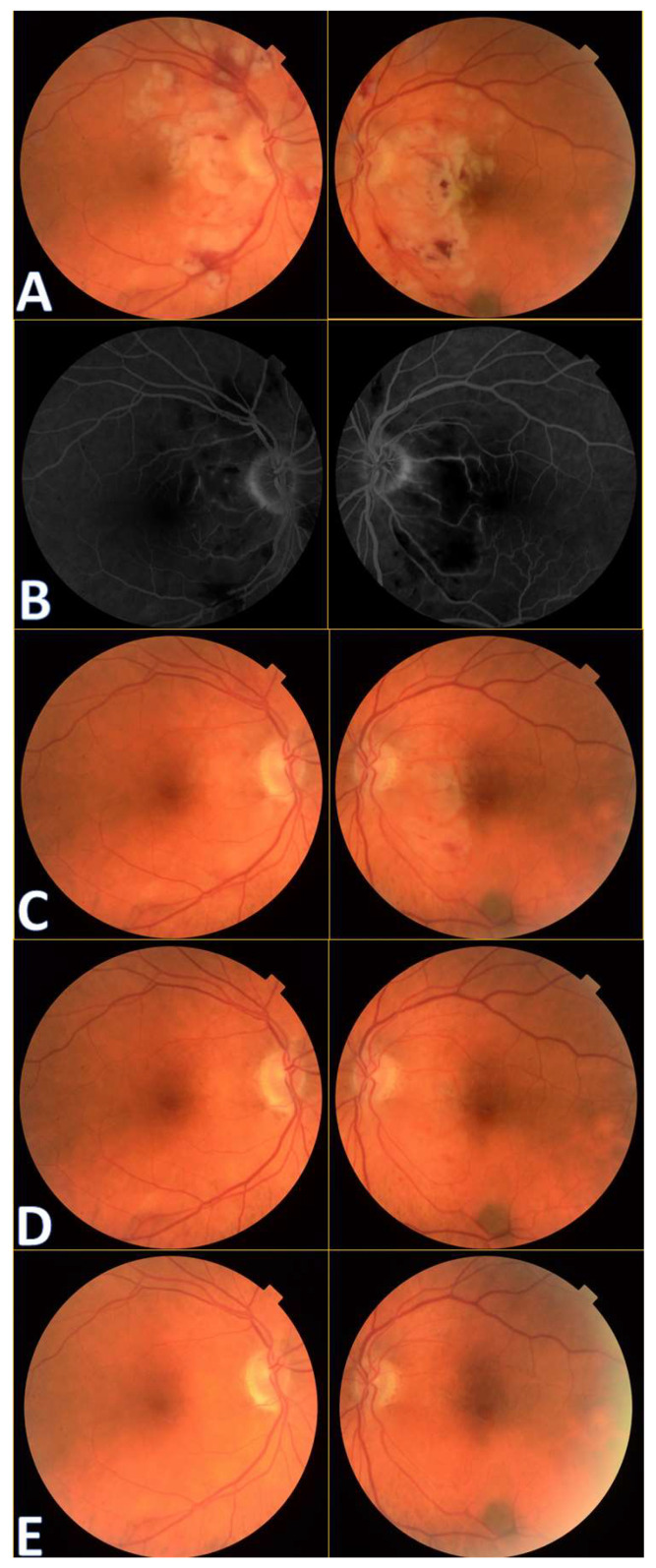
Colour fundus photography of the patients with Purtscher retinopathy shows cotton wool spots in the papillo-macular bundle region, retinal hemorrhages, and Purtscher flecken at initial presentation (**A**). A benign choroidal nevus can also be noticed bordering the inferotemporal vascular arcade in the left eye.

**Figure 2:**
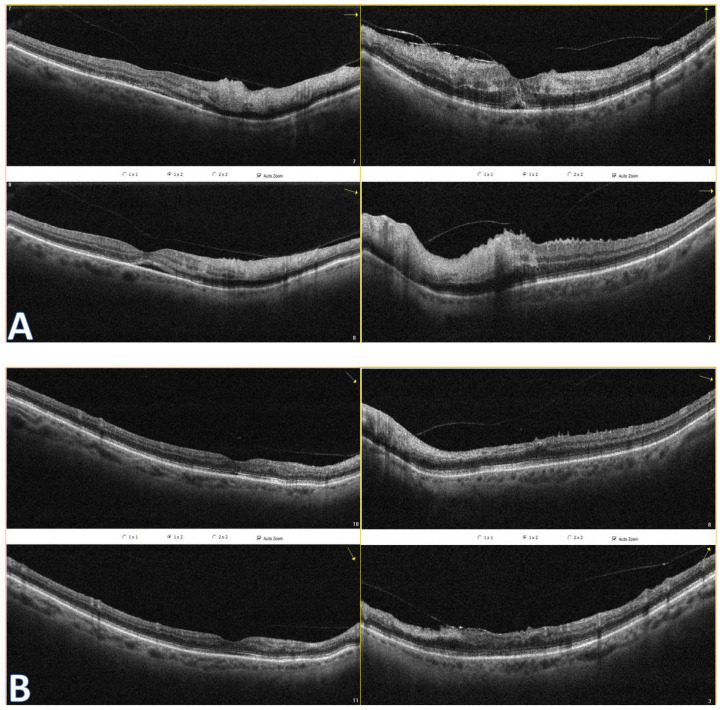
Optical coherence tomography of both eyes show hyperreflectivity of the inner retinal layers (**A**). Subretinal fluid is present. At follow-up (**B**), OCT reveals disorganization of inner retinal layers (DRIL) to some extent and their atrophy. Even though foveolar morphology looks better in the right eye, there is a rupture in the ellipsoid zone (EZ) continuum, which could explain worse BCVA in this eye.

**Figure 3:**
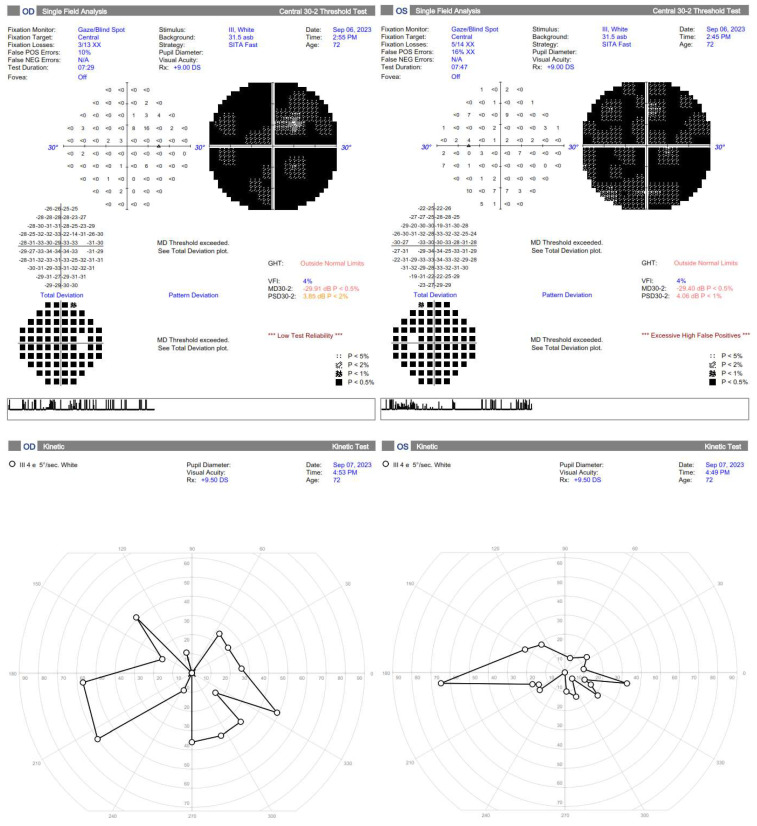

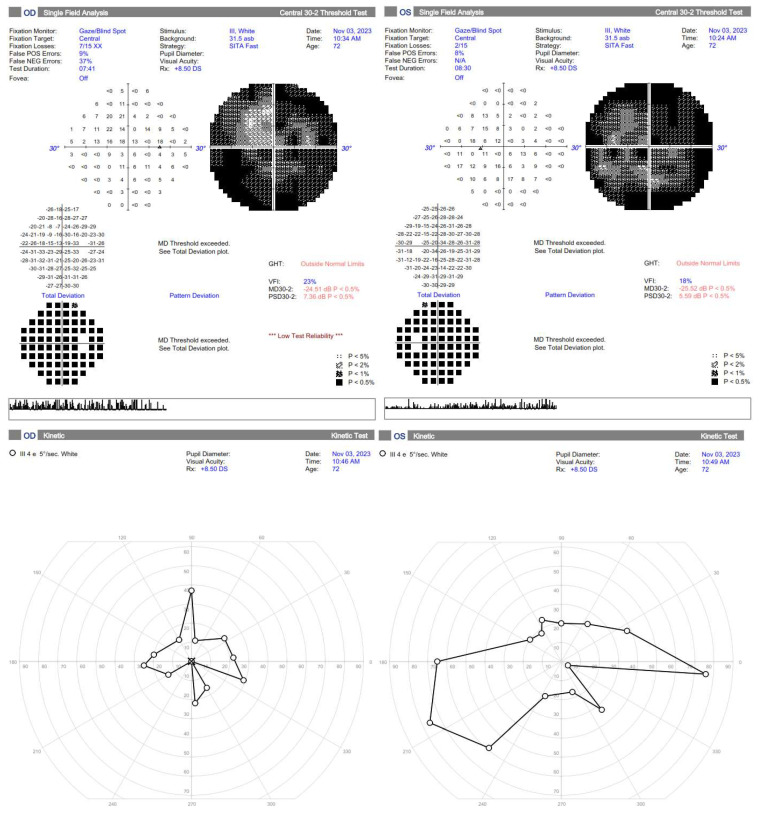
Standard automated perimetry is hardly interpretable in the first two months but shows general improvement, especially a much higher Visual Field Index and less absolute scotomas. The kinetic perimetry also demonstrates improvement in the left eye, but further restriction of the visual field in the right eye, probably due to failed reperfusion of the ischemic areas, resulting in retinal cells loss.

**Figure 4:**
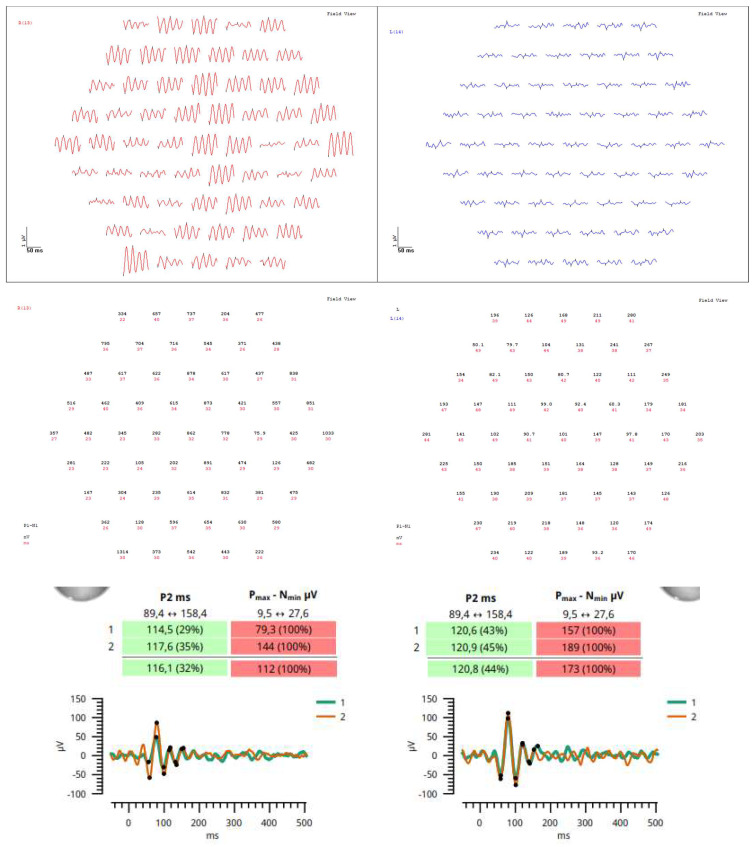
Due to poor reliability of the patient’s perimetry, multifocal electroretinography, and visually evoked potentials were performed at 6–month follow–up in order to better evaluate retinal physiology, showing normal time of electrophysiological responses but elevated amplitudes particularly on the right side. This may be due to extensive damage in the retinas, probably cells failing to communicate visual information and therefore firing stronger signals to help transmission. In this case, multimodal imaging, which is the use of two or more techniques to visualize the structure and assess the function of a target tissue, helped in the differential diagnosis and monitoring of Purtscher retinopathy.

## Data Availability

Data are available from the corresponding author upon reasonable request.
